# Cutaneous paragonimiasis due to triploid *Paragonimus westermani* presenting as a non-migratory subcutaneous nodule: a case report

**DOI:** 10.1186/1752-1947-8-346

**Published:** 2014-10-16

**Authors:** Makoto Kodama, Mayumi Akaki, Hiroyuki Tanaka, Haruhiko Maruyama, Eiji Nagayasu, Tetsuhiro Yokouchi, Yasuji Arimura, Hiroaki Kataoka

**Affiliations:** 1 Section of Oncopathology and Regenerative Biology, Department of Pathology, Faculty of Medicine, University of Miyazaki, 5200 Kihara, Kiyotake, Miyazaki, Miyazaki 889-1692, Japan; 2 Clinical Laboratory, University of Miyazaki Hospital, 5200 Kihara, Kiyotake, Miyazaki, Miyazaki 889-1692, Japan; 3 Division of Parasitology, Department of Infectious Diseases, Faculty of Medicine, University of Miyazaki, 5200 Kihara, Kiyotake, Miyazaki, Miyazaki 889-1692, Japan; 4 Yokouchi Dermatology and Plastic Surgery, 1445-69 Haranomae, Oshimacho, Miyazaki, Miyazaki 880-0824, Japan; 5 Division of Neurology, Respirology, Endocrinology and Metabolism, Department of Internal Medicine, Faculty of Medicine, University of Miyazaki, 5200 Kihara, Kiyotake, Miyazaki, Miyazaki 889-1692, Japan

**Keywords:** Subcutaneous nodule, *Paragonimus westermani*, Histopathology

## Abstract

**Introduction:**

Paragonimiasis is a food-borne infection caused by *Paragonimus* parasites. The lungs and pleura are the primary sites for the infection; however, ectopic infection can occur in other organs such as skin, liver and brain. It is difficult to make a diagnosis of ectopic paragonimiasis due to an ignorance of, and unfamiliarity with the disease. We report the case of a patient with subcutaneous paragonimiasis diagnosed by histopathological analysis and serological testing.

**Case presentation:**

A 39-year-old Chinese immigrant woman presented with a subcutaneous nodule in her left lower back. The nodule was initially suspected of lipoma and she was followed up on without any treatment. However, it gradually indurated and the nodule was resected surgically. A magnetic resonance imaging scan revealed a polycystic lesion with inhomogeneous low or high intensity on T1- or T2-weighted images, respectively. The rim of the lesion was enhanced after contrast enhancement, but the inside did not show high-signal intensity. A histological analysis of the surgically resected specimen revealed variable-sized tubulo-cystic structures. The cyst wall showed a granulomatous change with scant eosinophilic infiltration. A number of parasite ova were observed in the necrotic tissue inside the cysts, and a parasite body with a presumed oral sucker and reproductive organ was also detected, suggesting a trematode infection. A subsequent serological examination showed a positive reaction of her serum to the *Paragonimus westermani* antigen. No abnormal findings were found on her chest computed tomography scan. The diagnosis of subcutaneous paragonimiasis caused by *Paragonimus westermani* was made.

**Conclusions:**

We report a case presenting only as a non-migratory subcutaneous nodule without any pleuropulmonary lesion, which was initially suspected of lipoma but denied by magnetic resonance imaging scan results. The case was subsequently diagnosed as subcutaneous paragonimiasis from the results of histopathological analysis and serological testing.

## Introduction

Paragonimiasis is a food-borne infection caused by *Paragonimus* parasites, such as *Paragonimus westermani* and *Paragonimus skrjabini miyazakii*. Among the *Paragonimus* species, only *Paragonimus westermani* has a triploid variant, which can produce ova via parthenogenesis [1]. The primary organs for parasite infestation are the lungs and pleura, therefore most patients present with signs and symptoms involved in the lower respiratory tract and pleura such as cough, sputum, chest pain, dyspnea and pleural effusion. In some cases, ectopic infection occurs at unexpected sites such as skin, brain, liver and peritoneal cavity, due to erratic migration [1-3]. Ectopic paragonimiasis is difficult to confirm as a diagnosis because of its rarity and variable symptoms, which has caused an ignorance of, and unfamiliarity with of the disease. We report the case of a patient with subcutaneous paragonimiasis diagnosed using histopathological analysis and serological testing.

## Case presentation

**Figure 1:**
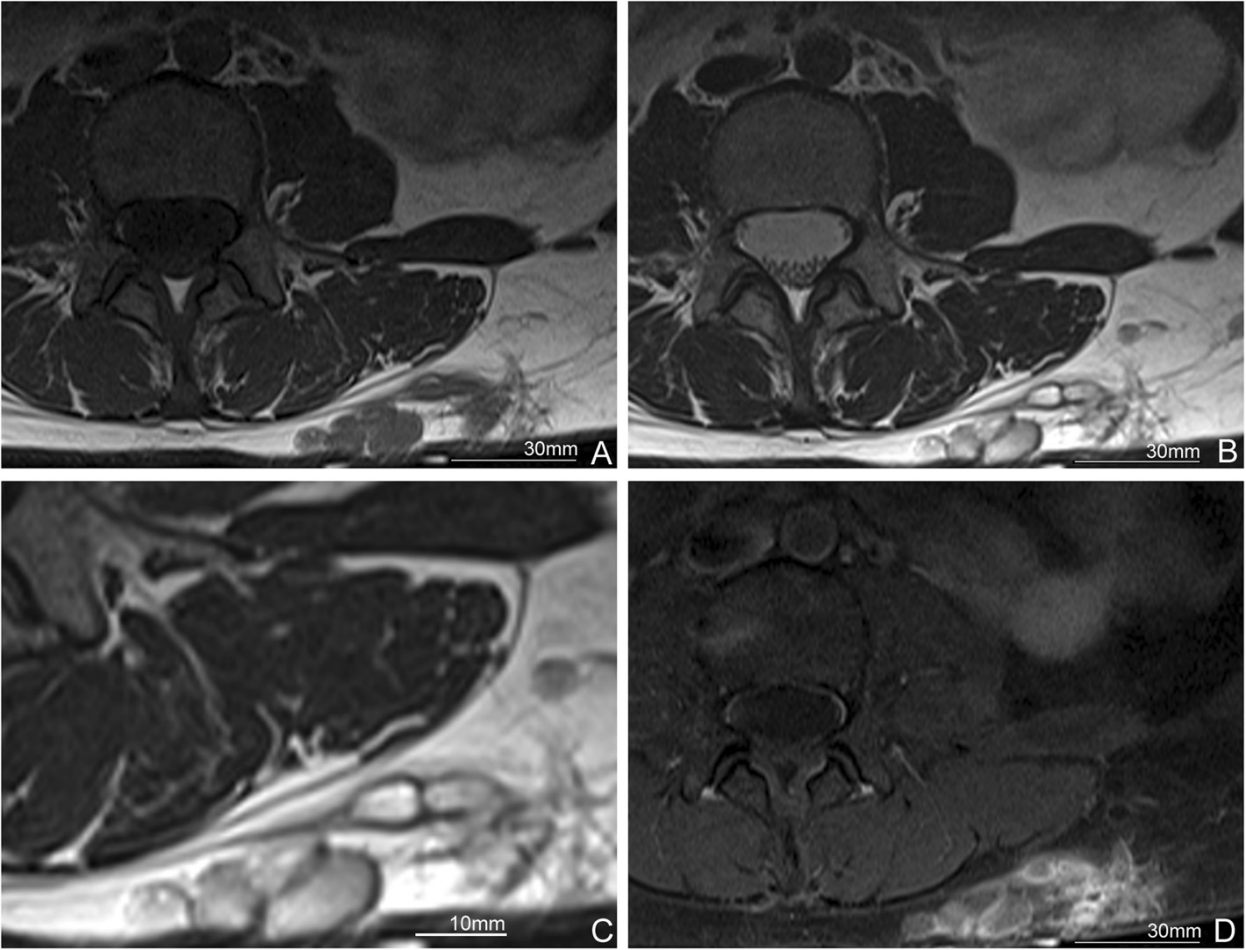
**Magnetic resonance imaging (MRI) findings. A**. Polycystic mass with inhomogeneous low intensity on T1-weighted image (T1WI). **B**. The mass with inhomogeneous high intensity on T2-weighted image (T2WI). **C**. The magnified image of the nodular lesion in **B**. **D**. Rim enhancement is seen but the inside of the mass shows no increment of density after contrast enhancement.

A 39-year-old Chinese immigrant woman had been aware of a subcutaneous nodule in her left lower back for a year and sought medical attention. In her past history, she had frequent opportunities to have been exposed to drunken crab (raw crab soaked in rice wine), especially before she emigrated in Japan seven years ago. Additionally, she had been a cigarette smoker in her twenties (5 cigarettes per day). No specific family history was addressed. The nodule was initially suspected of being soft tissue tumor, particularly lipoma, and followed up on for over a year without any treatment. However, the nodule gradually indurated and surgical resection was chosen as treatment. Her physical examination before the resection revealed neither fever nor abnormal pulmonary sounds. Her white blood cell (WBC) count was 6740/μL (reference range: 3500 to 8700/μL), and neither her eosinophil count nor serum immunoglobulin E (IgE) level was examined.She was suspected of having lipoma and a magnetic resonance imaging (MRI) scan was performed. A cystic lesion was found in the subcutaneous tissue on her left lower back, suggesting a closely-aggregated tortuous and inflected tubular architecture. In the lesion, inhomogeneous low-signal intensity was observed on a T1-weighted image (T1WI) (Figure 1A), and inhomogeneous high-signal intensity was observed on a T2-weighted image (T2WI) (Figure 1B and C). A contrast-enhanced MRI scan revealed a high-signal intensity was not detected in the inside while the rim of the nodule was enhanced (Figure 1D). No infiltrative lesion to the muscle layer or retroperitoneum was detected. The signal intensity of the contents of the lesion was similar to that of water, suggesting serous or mucinous fluid rather than blood. Based on these imaging findings, the preoperative diagnosis of the lesion was lymphatic vessel malformation or mucinous nodules. Subsequently, a surgical resection was performed.

**Figure 2:**
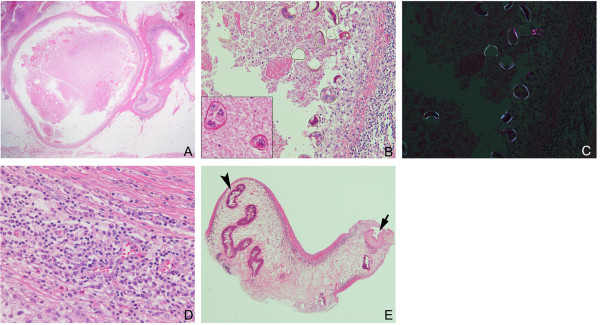
**Histopathological findings. A**. The lesion is composed of variable-sized tubulo-cystic structures embedded in subcutaneous tissue. Hematoxylin and eosin (HE) stain: 12.5×. **B**. A number of parasite ova are observed in the necrotic tissue inside the cystic structures. The shell thickness of ova is uneven and some are distorted. The ova contain several cells with round nuclei and eosinophilic cytoplasm, suggesting yolk cells (inset, 400×). HE stain: 200×. **C**. Egg shells of *Paragonimus westermani* are highlighted under polarized light in the same field as shown in **B**: 200×. **D**. The cyst wall with a granulomatous change and scant infiltration of eosinophils. HE stain: 400×. **E**. The section of the worm shows characteristics of trematode, the presumed oral sucker (arrow) and reproductive organ (arrow head). HE stain: 40×.

Macroscopically, the resected specimen was an abscess-like nodular lesion beneath the skin. On the cut surface, a nodular lesion consisted of creamy yellowish material containing a small hematoma-like fragment and was surrounded by a subcutaneous adipose tissue. Microscopically, this lesion was composed of one large tubulo-cystic structure and several small cysts (Figure 2A). The cyst walls showed a granulomatous change with scant eosinophilic infiltration (Figure 2D). A number of parasite ova were observed in necrotic debris inside the cystic structures. The ovular shell thickness was uneven and some were distorted. There were several cells with round nuclei and eosinophilic cytoplasm in the ova, suggesting yolk cells (Figure 2B). Each ovum was approximately 80×50μm in size. They were also intensely birefringent under polarized light (Figure 2C) [4]. These findings were compatible with those of the ova of *Paragonimus*, particularly *P. westermani*, in terms of size. No apparent operculum was detected in the hematoxylin and eosin (HE) stained sections. Furthermore, histologic analysis of the hematoma-like fragment in the main cyst revealed a parasite body with a presumed oral sucker and reproductive organ (Figure 2E). These finding are compatible with the characteristics of adult trematodes, raising the possibility of subcutaneous *Paragonimus* infection. In addition, only a single worm was found in the lesion, suggesting that this worm was a triploid form of *P. westermani* producing ova via parthenogenesis*,* not a diploid form of the *Paragonimus* species.

On serological examination using multiple-dot enzyme-linked immunosorbent assay with antigens of *P. westermani* and *P. skrjabini miyazakii*, which are two major species causing human paragonimiasis in Japan [5], serum obtained from the patient was positive for *P. westermani* antigen rather than *P. skrjabini miyazakii*. However, no abnormal finding was found in both the lungs and pleura in her chest computed tomography (CT) scan. We made a diagnosis of subcutaneous paragonimiasis by *P. westermani*. One month after the resection, the WBC count in her peripheral blood was 6450/μL with an eosinophil count of 200/μL, and her serum IgE value was 51.7IU/mL, which were all within normal range. No further medication or treatment was assigned as she did not have any other symptoms and no lesion was detected in her chest CT scan.

## Discussion

Paragonimiasis has been re-emerging in Japan due to the increase in immigrants and travelers from endemic areas, such as China and Thailand [5]. Nevertheless, paragonimiasis is still a rare disease in Japan, since annual new cases are around several dozen nationally [1]. Due to the rarity of the disease, it is significantly difficult to make a diagnosis of paragonimiasis and proper examinations are often not performed. For example, a case with bloody sputum, which is one of the typical clinical symptoms of pulmonary paragonimiasis, remained undiagnosed for three years [6]. A high eosinophil count in peripheral blood and an elevated serum IgE value are not specific findings; they are observed in around 80% of patients [5]. Even if a sputum test and biopsy are performed, the ova or body of *Paragonimus* parasites may not be detectable due to an insufficient amount of specimens [7].

Subcutaneous paragonimiasis is particularly rare compared to pleuropulmonary and the other ectopic paragonimiasis. According to a recent review of paragonimiasis cases in Japan, only 4.9% of the 384 cases presented with subcutaneous nodules [5]. In addition to the rarity, this case suffered from a lack of findings suggesting parasite infection: the subcutaneous mass was not migratory and an eosinophilic count in her peripheral blood and serum IgE value were unavailable. This caused difficulty in reaching the definitive diagnosis. In her histopathological examination the lesion was completely encapsulated by fibrosis, with a granulomatous change and scant eosinophilic infiltration. This may indicate a chronic phase of the infection, not an acute phase, which is in accordance with the long follow-up term before the resection. The resected tissue contained the parasite ova and body, which provided sufficient findings leading to the diagnosis of subcutaneous paragonimiasis. The following serological test confirmed an infection by *P. westermani*.

To the best of our knowledge, no report has been published regarding MRI findings of subcutaneous paragonimiasis in combination with histopathological examinations, although one case of subcutaneous paragonimiasis with a CT scan suggesting a localized abscess on the lower back has been reported [8]. On the other hand, MRI findings of liver and brain paragonimiasis have been reported, showing cystic lesions with low intensity signal in T1WI imaging and high intensity signal in T2WI imaging [9,10]. Subcutaneous nodules in this case presented similar MRI findings to those of the liver and brain cases, but additionally showed inhomogeneity of the content. A contrast-enhanced MRI scan revealed that the lesion was enhanced at the rim but not inside the nodules. Only a very few diseases, such as lymphatic vessel malformation, present similar MRI findings in subcutaneous tissue.

There are several infection sources of paragonimiasis. Freshwater crab (*Eriocheir japonica*, *Eriocheir sinensis* or *Geothelphusa dehaani)* is an intermediate host of *Paragonimus* and wild boar is a paratenic host. Humans become infected after eating these in a raw or undercooked state. Drunken crab and Japanese cuisine using freshwater crab or wild boar meat are thought to be the main infection sources in Japan [1,5,11]. A previous study suggested that the infection rate of freshwater crabs varies from 0 to 88%, according to the regions or seasons (the average infection rate is 17%) [12]. Paying careful attention to previous history related to freshwater crab and wild boar is important in making a diagnosis of paragonimiasis.

However, proper history-taking is often difficult, particularly in non-endemic areas of paragonimiasis. Since cutaneous and subcutaneous paragonimiasis lesions are relatively easy to reach, an excision biopsy for histopathological examination is useful. Even if a worm and/or ova are not observed in the lesion, findings such as an abscess with eosinophilic infiltration may be able to suggest parasite infection. In combination with serological testing, cutaneous and/or subcutaneous paragonimiasis can be diagnosed [13,14].

## Conclusions

We report a case of 39-year-old Chinese immigrant woman presenting with a subcutaneous nodule on her left lower back. Her MRI findings showed cystic lesions with inhomogeneous low intensity signal in a T1WI and high intensity signal in a T2WI. Rim enhancement was shown on a contrast-enhanced MRI scan. A histopathological examination revealed a granulomatous lesion associated with necrosis, ova and a body of a parasite suggesting trematode. A serological examination confirmed an infection of *P. westermani*. Together with the absence of pleuropulmonary lesion, this case was diagnosed as subcutaneous paragonimiasis.

## Consent

Written informed consent was obtained from the patient for publication of this case report and any accompanying images. A copy of the written consent is available for review by the Editor-in-Chief of this journal.

## Abbreviations

CT: Computed tomography; HE: Hematoxylin and eosin; IgE: Immunoglobulin E; MRI: Magnetic resonance imaging; T1WI: T1-weighted image; T2WI: T2-weighted image; WBC: White blood cell.

## Competing interests

The authors declare that they have no competing interests.

## Authors’ contributions

MK and MA were involved in acquisition of data and drafting the manuscript, and equally contributed to this study. HT designed and organized this study. HM guided the diagnosis by parasitological, histopathological and serological examinations. EN helped in parasitological conception. TY performed dermatological aspects of this study. YA performed respirological aspects of this study. HK revised the manuscript critically for important intellectual content and supported financially. All authors read and approved the final manuscript.

## Authors’ information

MK’s current address is Department of Human Pathology, Tokyo Medical and Dental University Graduate School, 1-5-45 Yushima, Bunkyo-ku, Tokyo 113–8510, Japan.
